# Supplementary material for: Pore engineering as a general strategy to improve protein-based enzyme nanoreactor performance
Source: bioRxiv. 2024 May 2:2024.05.02.592161. Preprint. [Version 1] doi: 10.1101/2024.05.02.592161 (PMC11092584; doi:10.1101/2024.05.02.592161)
Supplement: Supplement 1 [file media-1.pdf]

Supplementary Information

Pore engineering as a general strategy to improve protein-based enzyme nanoreactor performance

Seokmu Kwon[1], Michael P. Andreas[2], and Tobias W. Giessen[2]*
[1]Department of Chemical Engineering, University of Michigan, Ann Arbor, MI, USA
[2]Department of Biological Chemistry, University of Michigan Medical School, Ann Arbor, MI, USA
*correspondence: tgiessen@umich.edu

Table of Contents

Supplementary Fig. 1. SDS-PAGE gels of all proteins used in this study. 2
Supplementary Fig. 2. Additional negative-stain TEM micrographs 3
Supplementary Fig. 3. Cryo-EM processing of Mx_pmut T=1 shells 4
Supplementary Fig. 4. Cryo-EM processing of Mx_pmut T=3 and T=4 shells 5
Supplementary Fig. 5. Local resolution analysis of Mx_pmut shells 6
Supplementary Fig. 6. Dynamic light scattering analysis and thermal ramps 7
Supplementary Fig. 7. Trypsin and bacterial cell lysate incubations 10
Supplementary Fig. 8. Activity analysis of co-encapsulated PTDH 12
Supplementary Fig. 9. Activity analysis of co-encapsulated AdhD 13
Supplementary Table 1. Sequences of constructs used in this study 14
Supplementary Table 2. DNA primers used in this study 19
Supplementary Table 3. Cryo-EM data collection and refinement statistics 20

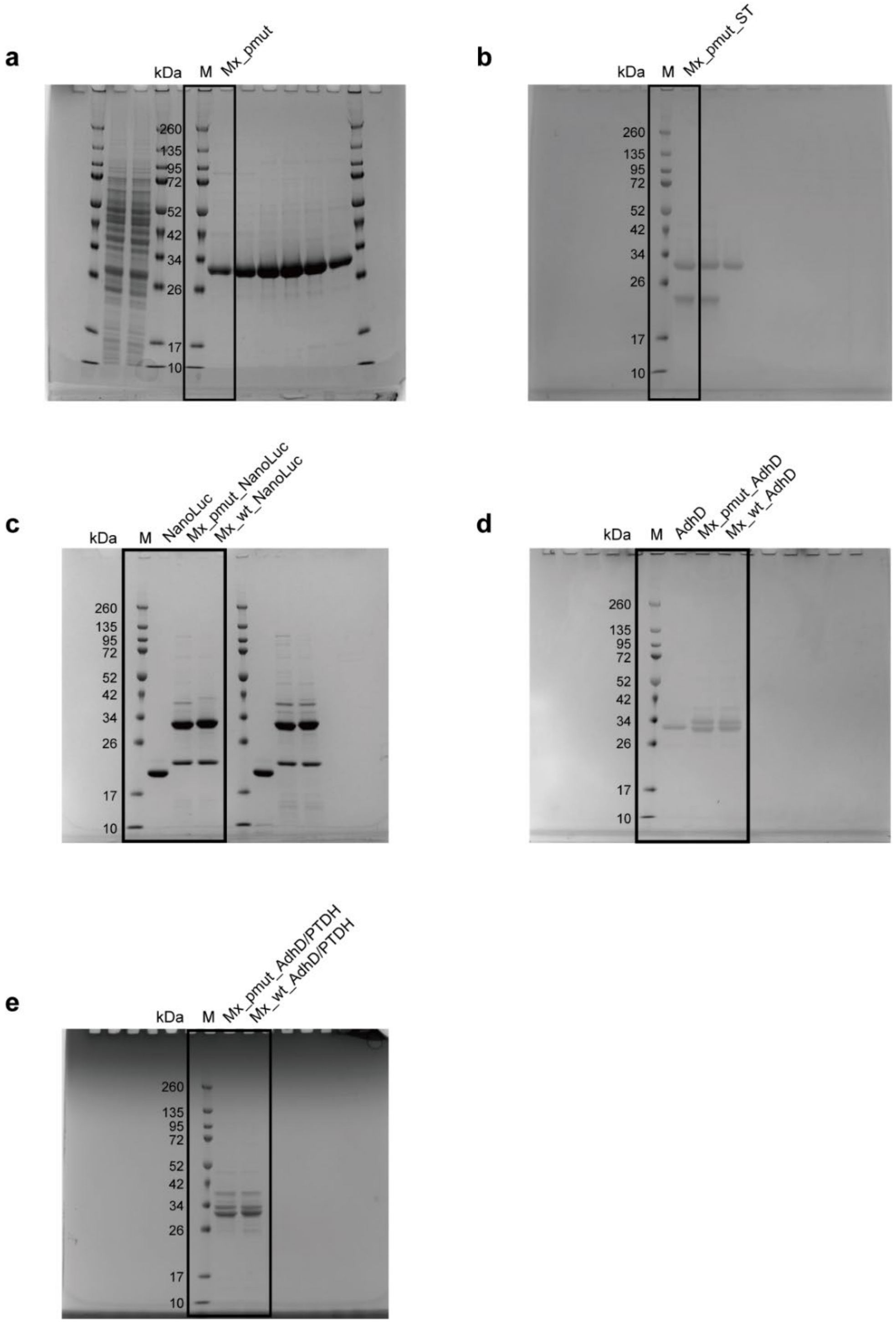

Supplementary Fig. 1. Full uncropped SDS-PAGE gels of all purified proteins used in this study. **a**, Mx_pmut. **b**, SNAP-tag (ST)-loaded Mx_pmut. **c**, proteins and nanoreactors for NanoLuc experiments. **d**, proteins and nanoreactors for AdhD experiments. **e**, proteins and nanoreactors used for cofactor recycling experiments.

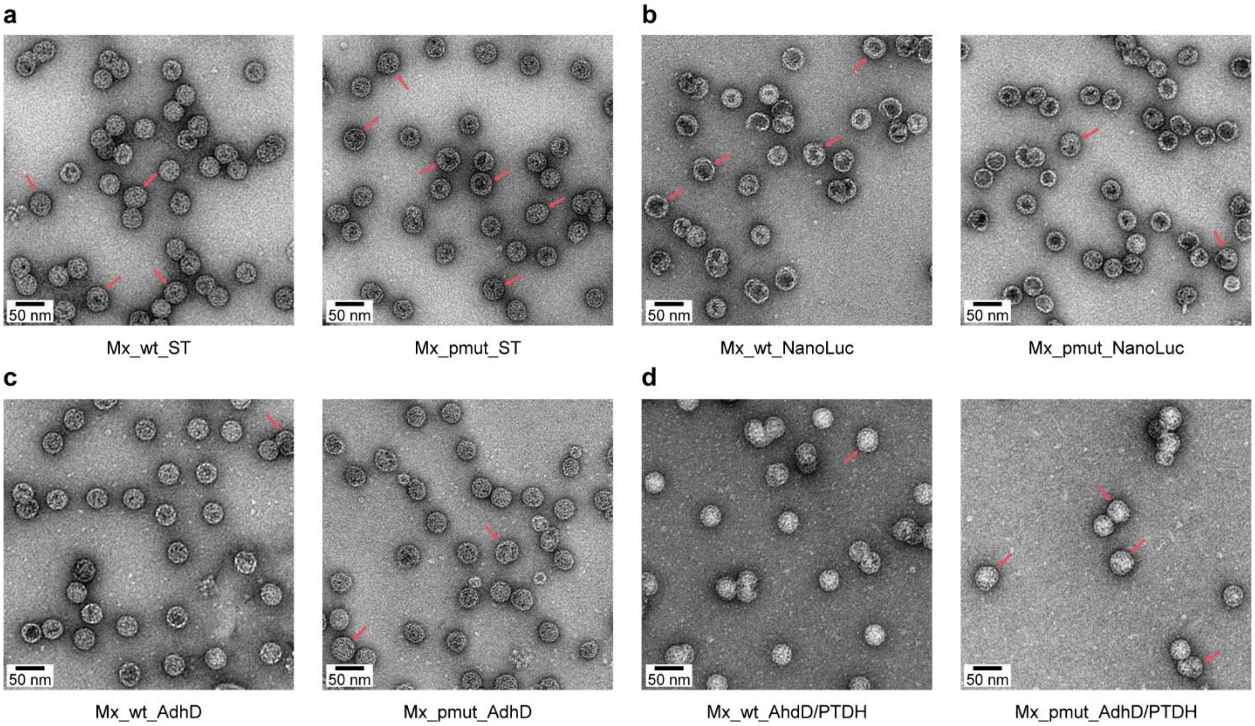

Supplementary Fig. 2. Negative-stain TEM micrographs of Mx_pmut- and Mx_wt-based-nanoreactors. **a**, SNAP-tag (ST) cargo. **b**, NanoLuc cargo. **c**, AdhD cargo. **d**, AdhD/PTDH cargo. Likely T=4 shells are highlighted with red arrows.

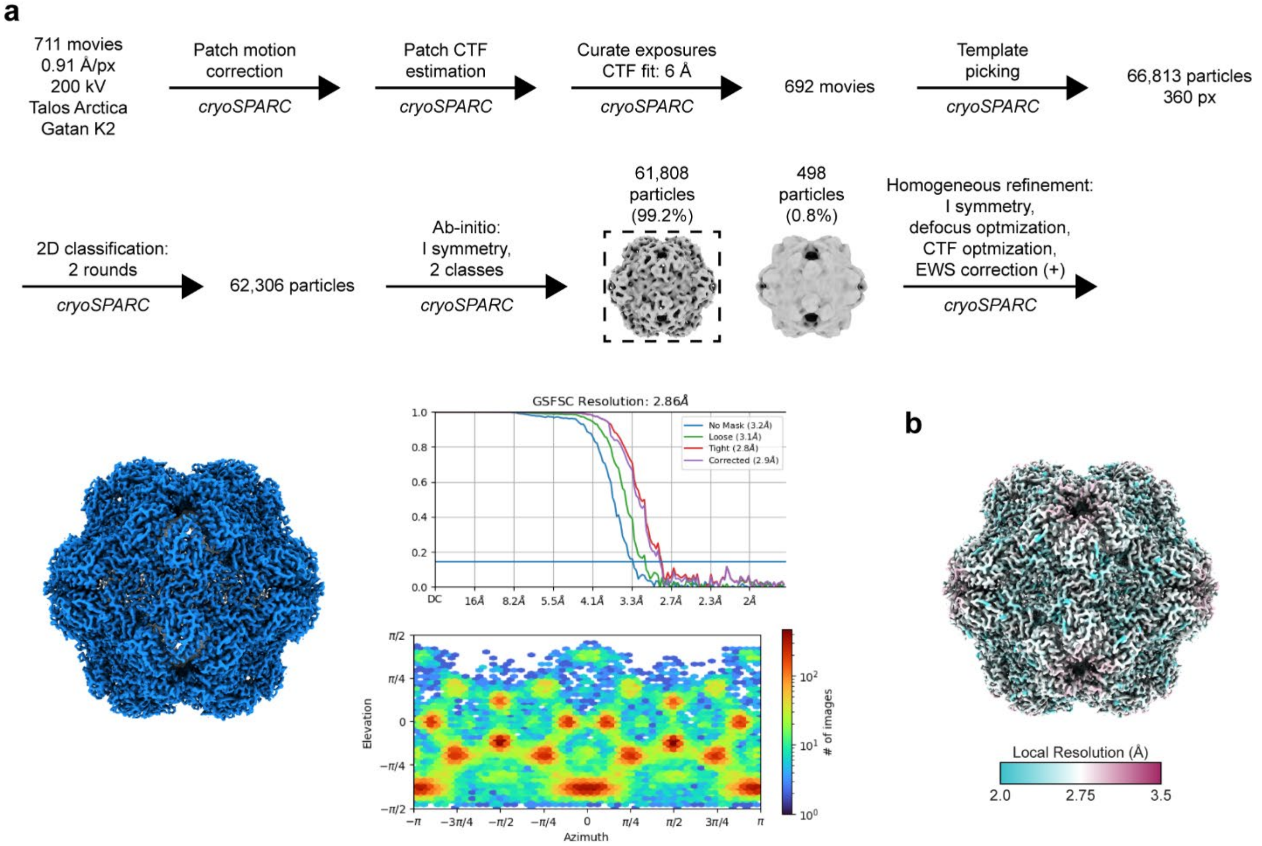

Supplementary Fig 3. Cryo-EM processing and analysis of Mx_pmut T=1 shells. a, Cryo-EM data processing workflow for the Mx_pmut T=1 shell without cargo. GSFSC: gold-standard Fourier shell correlation. Angular distribution of particles shown below the GSFSC plot. **b**, Local resolution analysis of the Mx_pmut T=1 cryo-EM density.

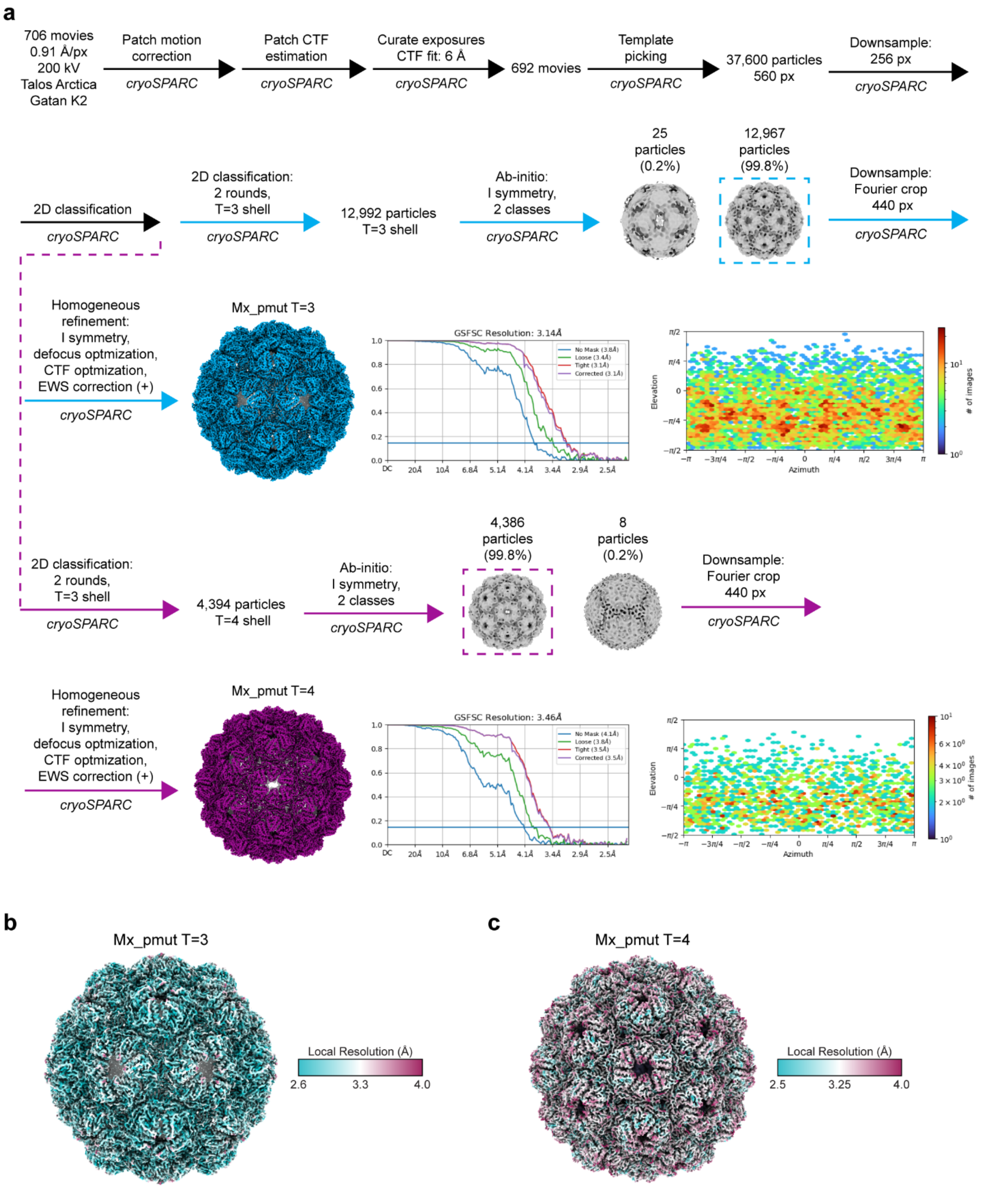

Supplementary Fig. 4. Cryo-EM processing and analysis of Mx_pmut T=3 and T=4 shells. **a**, Cryo-EM data processing workflow for Mx_pmut T=3 (cyan) and Mx_pmut T=4 (magenta). Angular distribution of particles shown below the GSFSC plots. **b**, Local resolution analysis of the Mx_pmut T=3 cryo-EM density. **c**, Local resolution analysis of the T=4 cryo-EM density.

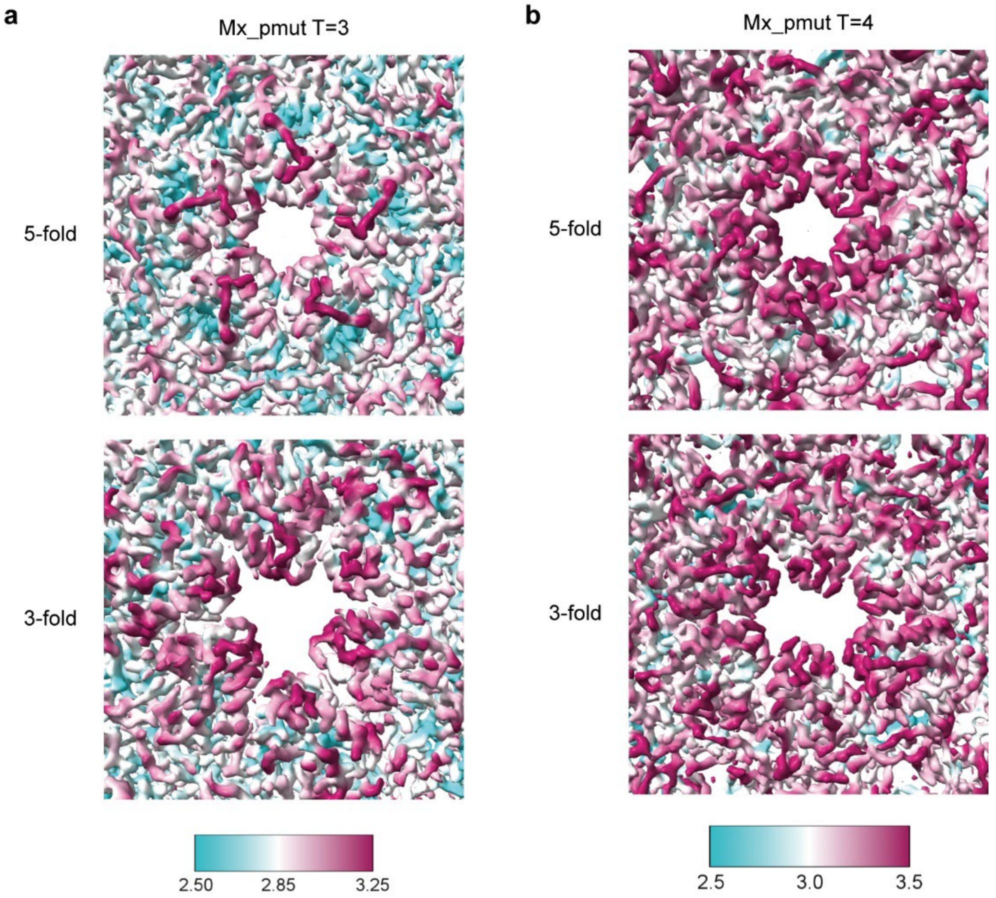

Supplementary Fig. 5. Local resolution analysis of Mx_pmut T=3 and T=4 pores. a, Mx_pmut T=3 5- and 3-fold pores. Local resolution scale shown below in Å. **b**, Mx_pmut T=4 5- and 3-fold pores. Local resolution scale shown below in Å.

a

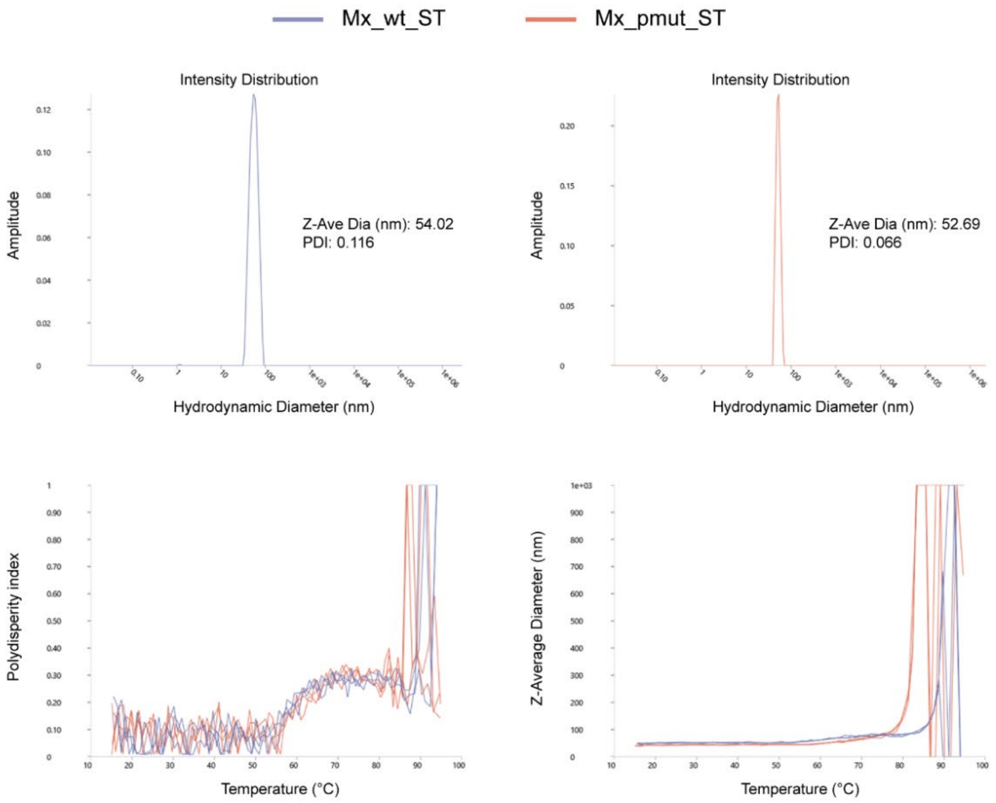
Mx_wt_ST
Mx_pmut_ST
Intensity Distribution
Amplitude
Hydrodynamic Diameter (nm)
Z-Ave Dia (nm): 54.02
PDI: 0.116
Intensity Distribution
Amplitude
Hydrodynamic Diameter (nm)
Z-Ave Dia (nm): 52.69
PDI: 0.066
Polydisperity index
Temperature (°C)
Z-Average Diameter (nm)
Temperature (°C)

b

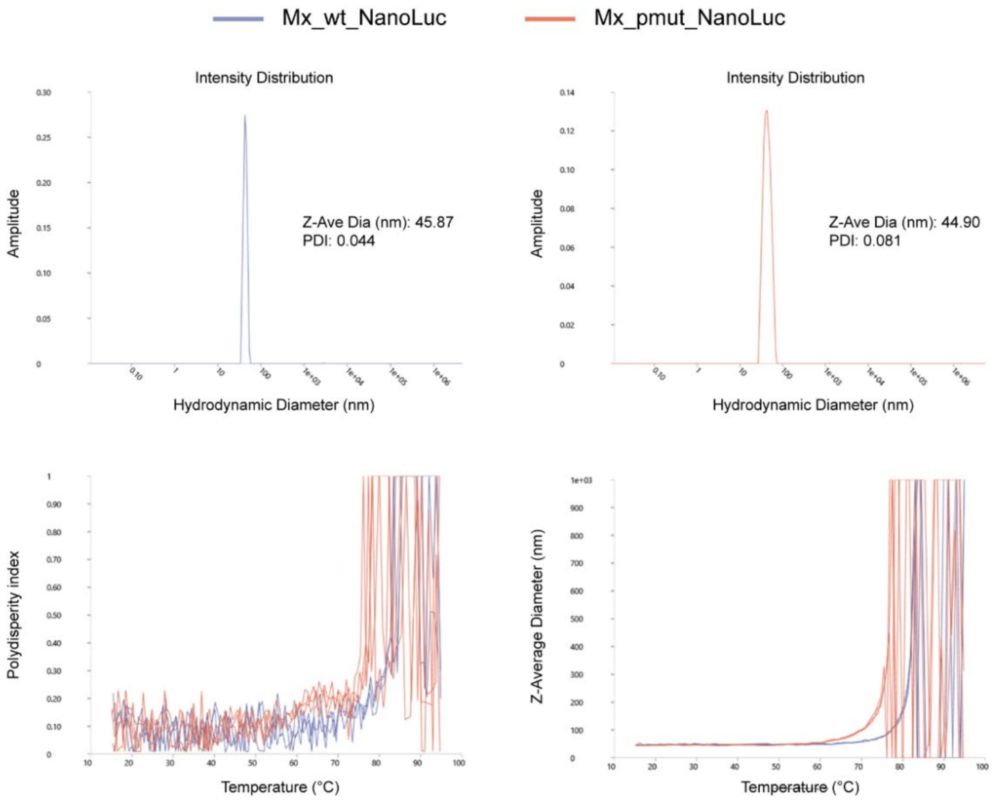
Mx_wt_NanoLuc
Mx_pmut_NanoLuc
Intensity Distribution
Amplitude
Hydrodynamic Diameter (nm)
Z-Ave Dia (nm): 45.87
PDI: 0.044
Intensity Distribution
Amplitude
Hydrodynamic Diameter (nm)
Z-Ave Dia (nm): 44.90
PDI: 0.081
Polydisperity index
Temperature (°C)
Z-Average Diameter (nm)
Temperature (°C)

c

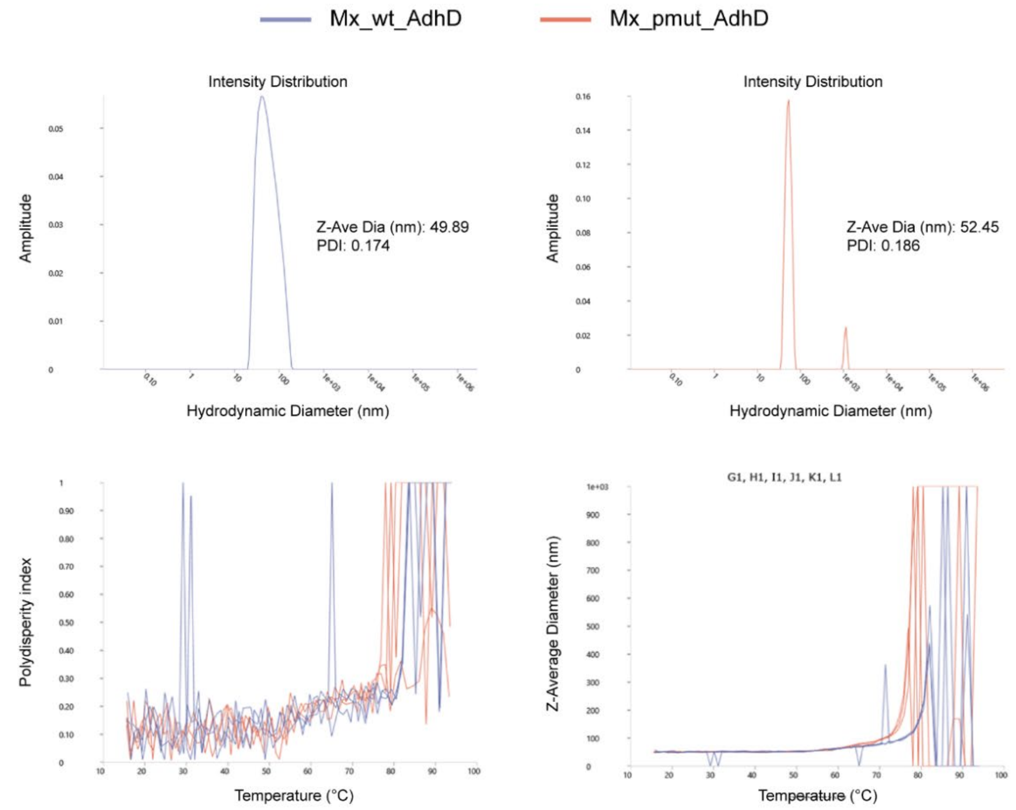

Mx_wt_AdhD
Mx_pmut_AdhD
Intensity Distribution
Amplitude
Hydrodynamic Diameter (nm)
Z-Ave Dia (nm): 49.89
PDI: 0.174
Intensity Distribution
Amplitude
Hydrodynamic Diameter (nm)
Z-Ave Dia (nm): 52.45
PDI: 0.186
Polydispersity index
Temperature (°C)
G1, H1, I1, J1, K1, L1
Z-Average Diameter (nm)
Temperature (°C)

d

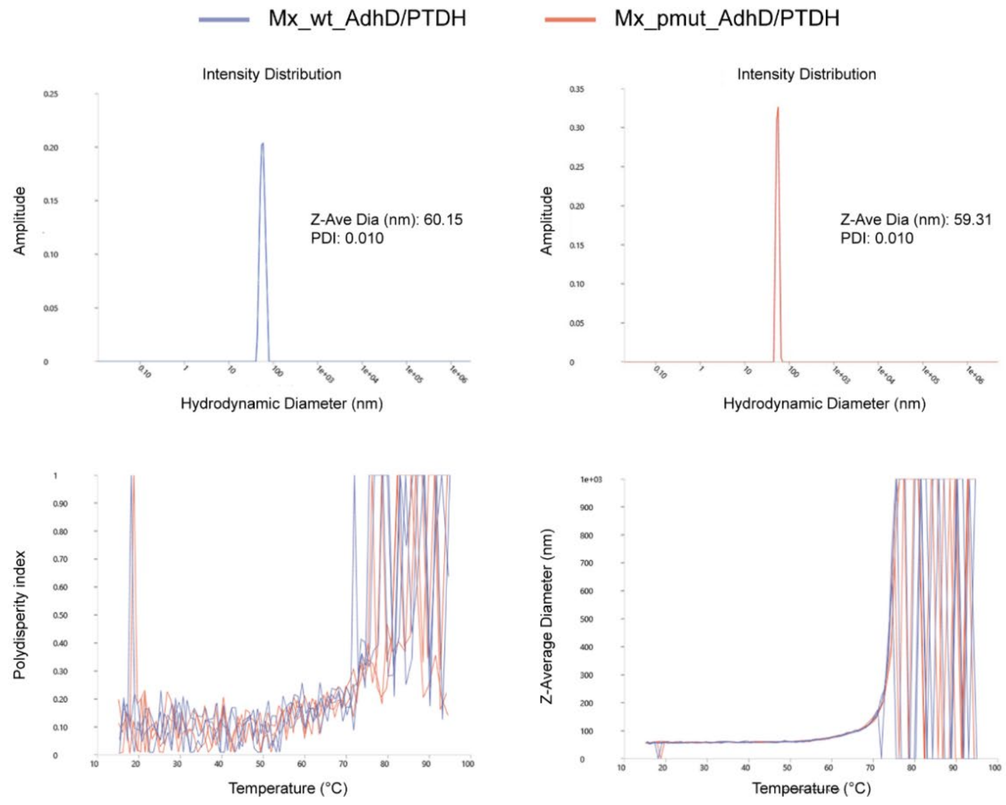

Mx_wt_AdhD/PTDH
Mx_pmut_AdhD/PTDH
Intensity Distribution
Amplitude
Hydrodynamic Diameter (nm)
Z-Ave Dia (nm): 60.15
PDI: 0.010
Intensity Distribution
Amplitude
Hydrodynamic Diameter (nm)
Z-Ave Dia (nm): 59.31
PDI: 0.010
Polydispersity index
Temperature (°C)
Z-Average Diameter (nm)
Temperature (°C)

Supplementary Fig. 6. Dynamic light scattering (DLS) analyses coupled with thermal ramps for Mx_wt- and Mx_pmut-based nanoreactors. **a**, SNAP-tag (ST) cargo. **b**, NanoLuc cargo. **c**, AdhD cargo. **d**, AdhD/PTDH cargo. Z-average diameters with polydispersity index (PDI) values for each nanoreactor system are shown (top left and right). Temperature was increased from 15°C to 95°C at 1°C/min while measuring PDI (bottom left) and Z-average diameter (bottom right). There was no meaningful thermal stability difference between Mx_wt- and Mx_pmut-based nanoreactors.

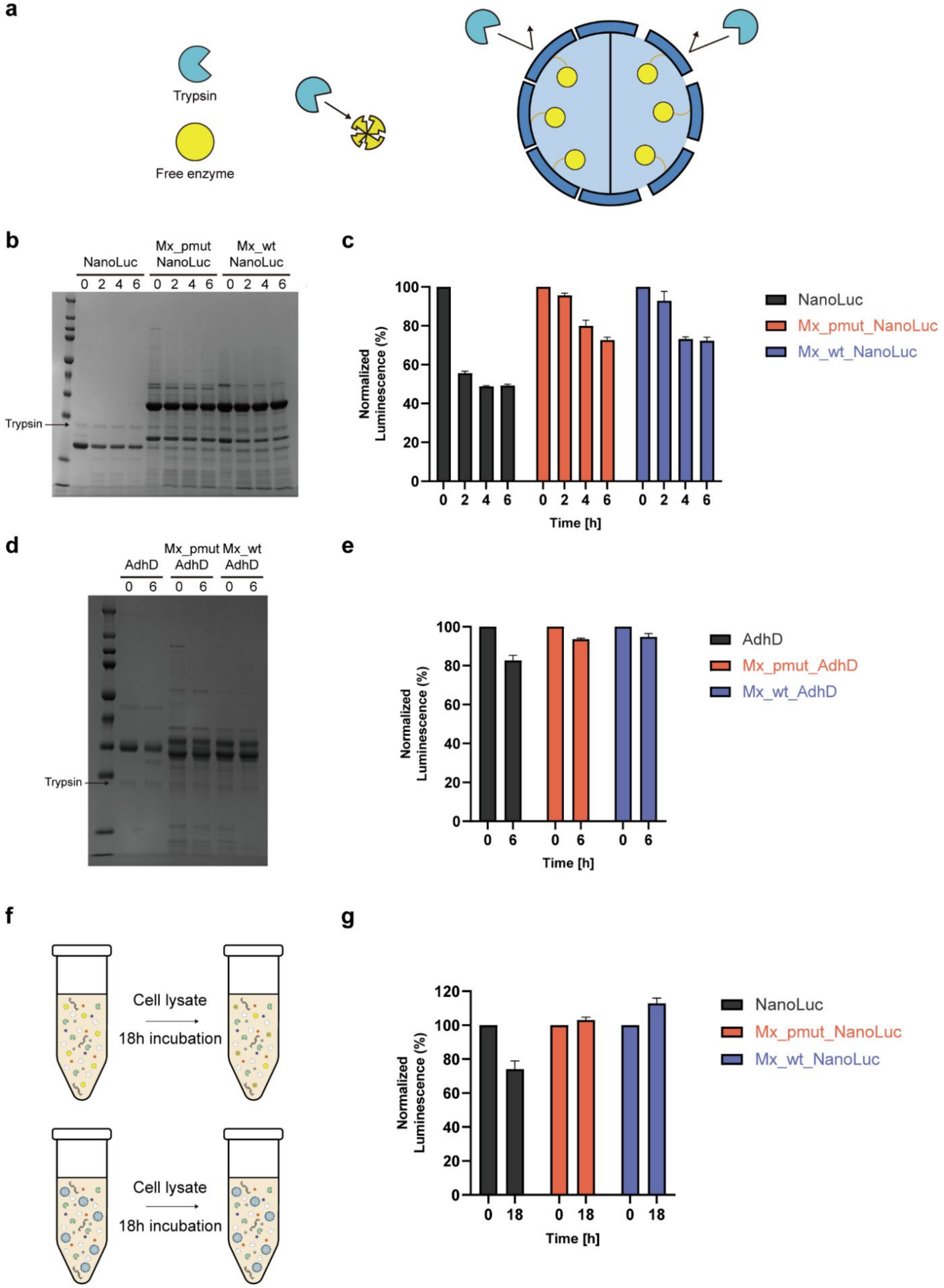
a
Trypsin
Free enzyme
b
NanoLuc
Mx_pmut NanoLuc
Mx_wt NanoLuc
0 2 4 6 0 2 4 6 0 2 4 6
Trypsin
c
Normalized Luminescence (%)
Time [h]
NanoLuc
Mx_pmut_NanoLuc
Mx_wt_NanoLuc
d
AdhD
Mx_pmut AdhD
Mx_wt AdhD
0 6 0 6 0 6
Trypsin
e
Normalized Luminescence (%)
Time [h]
AdhD
Mx_pmut_AdhD
Mx_wt_AdhD
f
Cell lysate
18h incubation
Cell lysate
18h incubation
g
Normalized Luminescence (%)
Time [h]
NanoLuc
Mx_pmut_NanoLuc
Mx_wt_NanoLuc

Supplementary Fig. 7. Trypsin and bacterial cell lysate incubations. a, Schematic illustrating the susceptibility of free enzymes to trypsin and the protective effect of Mx_pmut and Mx_wt shells. **b**, SDS-PAGE analysis showing the proteolytic resistance of Mx_wt- and Mx_pmut-encapsulated NanoLuc compared to free NanoLuc upon trypsin exposure. Band intensity for free NanoLuc markedly reduced following a 2 h incubation period while Mx_wt- and Mx_pmut-encapsulated NanoLuc exhibited little loss of band intensity over the examined time course. **c**, Enzymatic activity of trypsin-incubated free and Mx_wt- and Mx_pmut-encapsulated NanoLuc at given time points normalized by the activity before incubation. The activity of encapsulated NanoLuc was substantially more retained than that of free NanoLuc. **d**, SDS-PAGE analysis showing the proteolytic resistance of Mx_wt- and Mx_pmut-encapsulated AdhD compared to free AdhD upon trypsin exposure. Compared to NanoLuc, free AdhD was more resistant to trypsin exposure. **e**, Normalized enzymatic activity of trypsin-incubated free and Mx_wt- and Mx_pmut-encapsulated AdhD after 6 h of incubation. **f**, Schematic illustrating the susceptibility of free enzymes to harsh bacterial lysate conditions containing various proteases and reactive molecules and the protective effect of enzyme encapsulation. **g**, Normalized enzymatic activity of bacterial lysate-incubated free and Mx_wt- and Mx_pmut-encapsulated NanoLuc after 18 h of incubation. All error bars represent standard errors from three independent experiments.

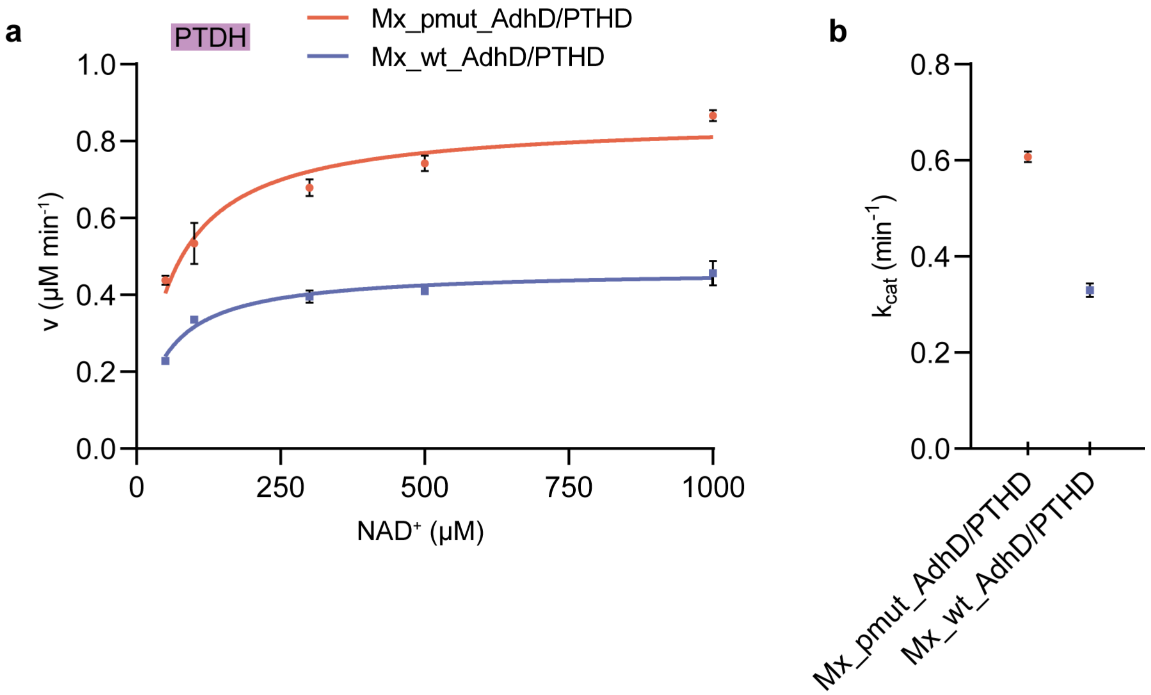

Supplementary Fig. 8. Activity analysis of co-encapsulated PTDH. a, Saturation kinetics analysis of PTDH in Mx_pmut- and Mx_wt-based AdhD/PTDH co-encapsulation nanoreactors. **b**, Comparison of turnover numbers (k_{cat}) for PTDH in Mx_pmut- and Mx_wt-based AdhD/PTDH co-encapsulation nanoreactors. All error bars represent standard error of three independent experiments.

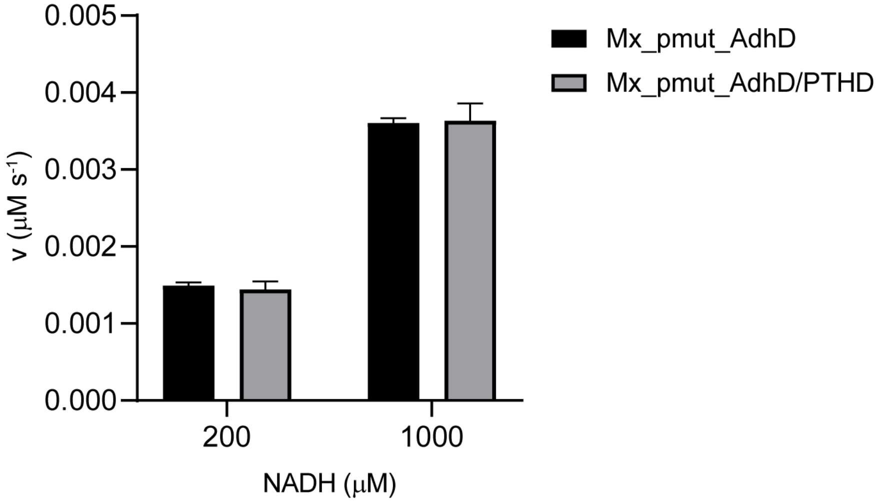

Supplementary Fig. 9. Activity analysis of co-encapsulated AdhD. AdhD activity assay examining the impact of co-encapsulation on AdhD activity compared to AdhD-only nanoreactors. For both nanoreactors, final concentrations of encapsulated AdhD in the reaction mixture was 15 nM and the AdhD activity was measured with 200 μM and 1000 μM NADH. There was no difference in AdhD activity demonstrating that AdhD activity was not negatively affected by co-encapsulation.

Supplementary Table 1. DNA and amino acid sequences of proteins used in this study.

Construct name	DNA sequence	Protein sequence
Mx_wt	ATGCCAGATTTTTTGGGTCACGCAGAAAAC<br>CCACTCCGGGAGGAAGAGTGGGCTCGCCT<br>TAACGAGACAGTTATTCAGGTTGCCCGTCG<br>TTCGCTTGTGGGGCGTCGGATTCTTGATAT<br>TTATGGCCCTTTGGGCGCAGGCGTCCAAAC<br>AGTACCATATGACGAATTTCAGGGTGTGAG<br>CCCAGGCGCAGTAGACATCGTCGGGGAAC<br>AAGAAACTGCTATGGTCTTCACCGACGCCC<br>GGAAGTTCAAAACTATCCCTATCATTTACAA<br>AGACTTCCTCCTTCATTGGCGTGACATCGA<br>AGCTGCGCGCACGCATAATATGCCTCTTGA<br>TGTAAGTGCGGCCGCCGGTGCAGCTGCCC<br>TTTGCGCCCAGCAAGAGGATGAGCTTATCT<br>TCTATGGCGACGCTCGGCTCGGGTATGAA<br>GGCCTTATGACGGCGAACGGTCGGCTGAC<br>TGTTCCATTAGGTGACTGGACTTCCCCGGG<br>TGGCGGCTTCCAGGCCATTGTCGAAGCCA<br>CTCGTAAGTTAAACGAACAAGGCCACTTTG<br>GTCCATACGCTGTTGTGCTGTCACCTCGCT<br>TATATTCCCAGTTACATCGGATTTACGAAAA<br>AACAGGGGTCTTAGAGATCGAGACAATTCG<br>CCAGCTCGCCTCAGATGGTGTTTATCAGTC<br>GAATCGGTTACGGGGTGAGAGTGGCGTCG<br>TGGTCTCTACAGGCCGTGAAAACATGGATT<br>TAGCGGTGAGTATGGATATGGTTGCAGCCT<br>ACTTAGGGGCATCCCGGATGAATCACCCTT<br>TTCGCGTACTGGAAGCCCTCCTTTTGCGCA<br>TCAAGCATCCTGACGCGATCTGTACGTTAG<br>AAGGTGCTGGTGCGACTGAGCGGCGC	MPDFLGHAENPLREEEWARLNETVIQVAR<br>RSLVGRRILDIYGPLGAGVQTVPYDEFQGV<br>SPGAVDIVGEQETAMVFTDARKFKTIPIIYK<br>DFLLHWRDIEAARTHNMPLDVSAAAGAAAL<br>CAQQEDELIFYGDARLGYEGLMTANGRLT<br>VPLGDWTSPGGGFQAIVEATRKLNEQGHF<br>GPYAVVLSPRLYSQLHRIYEKTGVLEIETIR<br>QLASDGVYQSNRLRGESGVVVSTGRENM<br>DLAVSMDMVAAYLGASRMNHPFRVLEALL<br>LRIKHPDAICTLEGAGATERR
Mx_pmut	ATGCCAGATTTTTTGGGTCACGCAGAAAAC<br>CCACTCCGGGAGGAAGAGTGGGCTCGCCT<br>TAACGAGACAGTTATTCAGGTTGCCCGTCG<br>TTCGCTTGTGGGGCGTCGGATTCTTGATAT<br>TTATGGCCCTTTGGGCGCAGGCGTCCAAAC<br>AGTACCATATGACGAATTTCAGGGTGTGAG<br>CCCAGGCGCAGTAGACATCGTCGGGGAAC<br>AAGAAACTGCTATGGTCTTCACCGACGCCC<br>GGAAGTTCAAAACTATCCCTATCATTTACAA<br>AGACTTCCTCCTTCATTGGCGTGACATCGA<br>AGCTGCGCGCACGCATAATATGCCTCTTGA<br>TGTAAGTGCGGCCGCCGGTGCAGCTGCCC<br>TTTGCGCCCAGCAAGAGGATGAGCTTATCT<br>TCTATGGCGACGCTCGGCTCGGGTATGAA<br>GGCCTTATGACGGCGAACGGTCGGCTGAC<br>TGTTCCATTAGGTGACTGGACTTCCCCGGG<br>TGGCGGCTTCCAGGCCATTGTCGAAGCCA<br>CTCGTAAGTTAAACGAACAAGGCCACTTTG<br>GTCCATACGCTGTTGTGCTGTCACCTCGCT<br>TATATTCCCAGTTACATCGGGGTGGCGAGA<br>TCGAGACAATTCGCCAGCTCGCCTCAGATG<br>GTGTTTATCAGTCGAATCGGTTACGGGGTG<br>AGAGTGGCGTCGTGGTCTCTACAGGCCGT<br>GAAAACATGGATTTAGCGGTGAGTATGGAT<br>ATGGTTGCAGCCTACTTAGGGGCATCCCG<br>GATGAATCACCCTTTTCGCGTACTGGAAGC<br>CCTCCTTTTGCGCATCAAGCATCCTGACGC<br>GATCTGTACGTTAGAAGGTGCTGGTGCGAC<br>TGAGCGGCGC	MPDFLGHAENPLREEEWARLNETVIQVAR<br>RSLVGRRILDIYGPLGAGVQTVPYDEFQGV<br>SPGAVDIVGEQETAMVFTDARKFKTIPIIYK<br>DFLLHWRDIEAARTHNMPLDVSAAAGAAAL<br>CAQQEDELIFYGDARLGYEGLMTANGRLT<br>VPLGDWTSPGGGFQAIVEATRKLNEQGHF<br>GPYAVVLSPRLYSQLHRGGEIETIRQLASD<br>GVYQSNRLRGESGVVVSTGRENMDLAVS<br>MDMVAAYLGASRMNHPFRVLEALLLRIKHP<br>DAICTLEGAGATERR
SNAP-tag_His	ATGGGCCCAGGGAGTGACAAGGATTGCGA<br>GATGAAGCGGACCACTTTGGACTCACCGCT<br>CGGGAAGCTTGAATTGTCCGGTTGCGAGC<br>AAGGGTTACACGAGATTATCTTTTTAGGTAA<br>GGGGACGAGTGCTGCGGACGCAGTAGAAG	MGPGSDKDCEMKRTTLDSPLGKLELSGCE<br>QGLHEIIFLGKGTSAADAVEVPAPAAVLGG<br>PEPLMQATAWLNAYFHQPEAIEEFPVPALH<br>HPVFQQESFTRQVLWKLLKVVKFGEVISYS<br>HLAALAGNPAATAAVKTALSGNPVPILIPCH

	TTCCTGCTCCTGCCGCGGTGTTGGGCGGT CCTGAACCGCTCATGCAAGCTACGGCGTG GCTCAACGCCTATTTTCACCAGCCGGAAGC TATCGAAGAGTTTCCGGTACCTGCATTGCA CCATCCTGTGTTCCAACAAGAGTCTTTCAC ACGCCAGGTCCTGTGGAAGTTATTAAAGGT TGTCAAGTTTGGCGAGGTCATCAGCTATAG TCACTTGGCGGCCCTCGCTGGTAATCCTGC TGCCACAGCCGCGGTAAAAACTGCACTCTC CGGTAACCCTGTACCGATTTTAATCCCGTG CCACCGTGTGGTTCAGGGGGATCTGGATG TCGGGGGCTACGAGGGGGGGTTAGCGGTT AAGGAATGGTTGCTCGCTCATGAAGGCCAT CGTTTGGGGAAACGGGGCGGGGGTTCAGG CGGGGGCAGTCATCACCACCATCACCAT	RVVQGDLDVGGYEGGLAVKEWLLAHEGH RLGKRGGGSGGGSHHHHHH
SNAP-tag_MxTP	ATGGGTCCAGGTAGTGATAAAGATTGCGAA ATGAAACGCACTACCTTAGATTCCCCGCTC GGTAAGTTGGAGTTGAGCGGGTGCGAACA AGGCTTGCATGAAATCATTTTTCTGGGTAA GGGCACGTCAGCAGCAGATGCAGTAGAAG TGCCAGCCCCGGCGGCTGTTCTGGGGGGC CCAGAGCCGCTGATGCAAGCTACCGCATG GCTCAATGCCTATTTTCACCAGCCTGAGGC TATCGAGGAGTTTCCTGTGCCTGCGCTTCA CCATCCGGTCTTCCAGCAGGAGTCGTTTAC TCGGCAGGTACTGTGGAAGCTGTTGAAGGT TGTCAAGTTTGGGGAAGTAATCTCATATAGT CACTTAGCCGCGTTAGCCGGTAATCCTGCG GCTACAGCCGCGGTGAAGACTGCTTTAAGC GGGAACCCAGTTCCTATCCTTATCCCATGC CACCGCGTGGTACAAGGCGACCTCGACGT GGGCGGGTATGAGGGTGGGTTGGCGGTCA AGGAATGGTTGCTGGCCCATGAAGGGCAT CGGTTGGGGAAACGTGGTGGGGGTAGTGG CGGGGGTAGCCCAGAAAAGCGCTTGACAG TAGGGTCTCTCCGGCGT	MGPGSDKDCEMKRTTLDSPLGKLELSGCE QGLHEIIFLGKGTSAADAVEVPAPAAVLGG PEPLMQATAWLNAYFHQPEAIEEFPVPALH HPVFQQESFTRQVLWKLLKVVKFGEVISYS HLAALAGNPAATAAVKTALSGNPVPILIPCH RVVQGDLDVGGYEGGLAVKEWLLAHEGH RLGKRGGGSGGGSPEKRLTVGSLRR
NanoLuc_His	ATGGTTTTCACCTTAGAAGATTTCGTGGGC GACTGGCGCCAGACTGCAGGTTACAATCTC GACCAAGTACTCGAGCAGGGTGGTGTCTC GAGCTTATTTCAAAATCTGGGCGTGAGTGT GACTCCGATTCAGCGTATCGTACTGTCCGG TGAGAACGGGCTGAAGATCGACATCCACGT GATCATCCCGTACGAAGGGCTCTCGGGCG ATCAGATGGGCCAAATTGAGAAAATCTTTAA GGTGGTTTACCCGGTTGATGACCATCATTT TAAAGTTATCCTGCATTACGGCACGCTTGT CATTGACGGCGTTACACCAAACATGATCGA CTACTTTGGGCGCCCGTACGAAGGGATCG CTGTGTTTGACGGGAAAAAAATCACCGTAA CGGGTACTCTCTGGAATGGTAATAAGATTA TCGACGAACGTTTAATCAATCCAGACGGCT CTCTCTTGTTCCGCGTCACTATCAATGGCG TAACAGGTTGGCGTTTATGTGAGCGTATCC TCGCCGGTGGTGGGAGCGGGGGCGGTTC ACATCACCACCATCACCAT	MVFTLEDFVGDWRQTAGYNLDQVLEQGG VSSLFQNLGVSVTPIQRIVLSGENGLKIDIHV IIPYEGLSGDQMGQIEKIFKVVYPVDDHHFK VILHYGTLVIDGVTPNMIDYFGRPYEGIAVF DGKKITVTGTLWNGNKIIDERLINPDGSLLF RVTINGVTGWRLCERILAGGGSGGGSHHH HHH
NanoLuc_MxTP	ATGGTTTTCACCTTAGAAGATTTCGTGGGC GACTGGCGCCAGACTGCAGGTTACAATCTC GACCAAGTACTCGAGCAGGGTGGTGTCTC GAGCTTATTTCAAAATCTGGGCGTGAGTGT GACTCCGATTCAGCGTATCGTACTGTCCGG TGAGAACGGGCTGAAGATCGACATCCACGT GATCATCCCGTACGAAGGGCTCTCGGGCG ATCAGATGGGCCAAATTGAGAAAATCTTTAA GGTGGTTTACCCGGTTGATGACCATCATTT TAAAGTTATCCTGCATTACGGCACGCTTGT CATTGACGGCGTTACACCAAACATGATCGA CTACTTTGGGCGCCCGTACGAAGGGATCG	MVFTLEDFVGDWRQTAGYNLDQVLEQGG VSSLFQNLGVSVTPIQRIVLSGENGLKIDIHV IIPYEGLSGDQMGQIEKIFKVVYPVDDHHFK VILHYGTLVIDGVTPNMIDYFGRPYEGIAVF DGKKITVTGTLWNGNKIIDERLINPDGSLLF RVTINGVTGWRLCERILAGGGSGGGSPEK RLTVGSLRR

	CTGTGTTTGACGGGAAAAAAATCACCGTAA CGGGTACTCTCTGGAATGGTAATAAGATTA TCGACGAACGTTTAATCAATCCAGACGGCT CTCTCTTGTTCCGCGTCACTATCAATGGCG TAACAGGTTGGCGTTTATGTGAGCGTATCC TCGCCGGTGGTGGGAGCGGGGGCGGTTC ACCTGAGAAACGCTTGACTGTTGGGTCTCT CCGGCGT	
AdhD_His	ATGAAGCGCGTTAACGCATTTAACGACCTG AAACGTATTGGGGATGATAAGGTTACTGCA ATTGGTATGGGGACATGGGGCATCGGGGG CCGTGAAACTCCTGACTATTCCCGCGACAA GGAGTCCATCGAAGCAATCCGGTATGGCTT GGAGCTCGGCATGAACCTTATTGACACGGC TGAATTTTATGGGGCGGGCCACGCAGAAG AGATTGTTGGTGAGGCTATCAAGGAATTTG AACGTGAGGACATTTTTATCGTGTCGAAGG TATGGCCGACACATTTTGGGTACGAAGAAG CAAAGAAAGCGGCCCGGGCCTCGGCCAAG CGCTTGGGTACTTATATTGATTTGTACTTAT TGCACTGGCCTGTTGACGATTTCAAAAAAA TTGAAGAGACCTTGCATGCCCTTGAAGACC TCGTCGACGAGGGGGTCATCCGTTACATC GGCGTTTCAAACTTCAACTTAGAACTTCTCC AACGGTCCCAGGAGGTGATGCGCAAATAC GAGATCGTAGCGAACCAAGTGAAGTATTCT GTAAAGGACCGGTGGCCAGAAACCACGGG TCTCCTGGATTACATGAAGCGGGAGGGCAT CGCTTTAATGGCTTACACGCCACTTGAGAA AGGTACTTTGGCGCGGAATGAATGTCTTGC GAAGATCGGGGAGAAATACGGTAAAACCG CGGCACAGGTAGCCCTCAACTATCTGATTT GGGAAGAAAATGTGGTGGCGATCCCTAAG GCATCTAATAAGGAACACTTGAAAGAGAAT TTCGGGGCCATGGGTTGGCGCCTTAGCGA AGAAGACCGCGAGATGGCTCGTCGCTGTG TTGGCGGCGGTAGTGGTGGTGGTTCTGGG GGTGGTAGCCATCACCACCATCACCAT	MKRVNAFNDLKRIGDDKVTAIGMGTWGIG GRETPDYSRDKESIEAIRYGLELGMNLIDTA EFYGAGHAEEIVGEAIKEFEREDIFIVSKVW PTHFGYEEAKKAARASAKRLGTYIDLYLLH WPVDDFKKIEETLHALEDLVDEGVIRYIGVS NFNLELLQRSQEVMRKYEIVANQVKYSVKD RWPETTGLLDYMKREGIALMAYTPLEKGTL ARNECLAKIGEKYGKTAAQVALNYLIWEEN VVAIPKASNKEHLKENFGAMGWRLSEEDR EMARRCVGGGSGGGSGGGSHHHHHH
AdhD_MxTP	ATGAAGCGCGTTAACGCATTTAACGACCTG AAACGTATTGGGGATGATAAGGTTACTGCA ATTGGTATGGGGACATGGGGCATCGGGGG CCGTGAAACTCCTGACTATTCCCGCGACAA GGAGTCCATCGAAGCAATCCGGTATGGCTT GGAGCTCGGCATGAACCTTATTGACACGGC TGAATTTTATGGGGCGGGCCACGCAGAAG AGATTGTTGGTGAGGCTATCAAGGAATTTG AACGTGAGGACATTTTTATCGTGTCGAAGG TATGGCCGACACATTTTGGGTACGAAGAAG CAAAGAAAGCGGCCCGGGCCTCGGCCAAG CGCTTGGGTACTTATATTGATTTGTACTTAT TGCACTGGCCTGTTGACGATTTCAAAAAAA TTGAAGAGACCTTGCATGCCCTTGAAGACC TCGTCGACGAGGGGGTCATCCGTTACATC GGCGTTTCAAACTTCAACTTAGAACTTCTCC AACGGTCCCAGGAGGTGATGCGCAAATAC GAGATCGTAGCGAACCAAGTGAAGTATTCT GTAAAGGACCGGTGGCCAGAAACCACGGG TCTCCTGGATTACATGAAGCGGGAGGGCAT CGCTTTAATGGCTTACACGCCACTTGAGAA AGGTACTTTGGCGCGGAATGAATGTCTTGC GAAGATCGGGGAGAAATACGGTAAAACCG CGGCACAGGTAGCCCTCAACTATCTGATTT GGGAAGAAAATGTGGTGGCGATCCCTAAG GCATCTAATAAGGAACACTTGAAAGAGAAT TTCGGGGCCATGGGTTGGCGCCTTAGCGA AGAAGACCGCGAGATGGCTCGTCGCTGTG TTGGCGGCGGTAGTGGTGGTGGTTCTGGG	MKRVNAFNDLKRIGDDKVTAIGMGTWGIG GRETPDYSRDKESIEAIRYGLELGMNLIDTA EFYGAGHAEEIVGEAIKEFEREDIFIVSKVW PTHFGYEEAKKAARASAKRLGTYIDLYLLH WPVDDFKKIEETLHALEDLVDEGVIRYIGVS NFNLELLQRSQEVMRKYEIVANQVKYSVKD RWPETTGLLDYMKREGIALMAYTPLEKGTL ARNECLAKIGEKYGKTAAQVALNYLIWEEN VVAIPKASNKEHLKENFGAMGWRLSEEDR EMARRCVGGGSGGGSGGGSPEKRLTVGS LRR

	GGTGGTAGCCCGGAGAAACGGCTCACTGT AGGGAGCCTGCGTCGT	
PTDH_His	ATGTTGCCTAAGCTTGTGATTACACATCGC GTACATGAGGAGATTCTGCAGTTATTGGCG CCTCATTGTGAGCTGATTACCAACCAGACA GATTCAACCCTCACGCGTGAAGAAATTTTA CGCCGTTGTCGTGACGCTCAAGCGATGAT GGCATTTATGCCGGACCGCGTTGATGCTGA CTTCTTACAAGCCTGTCCTGAACTGCGTGT AATCGGGTGCGCTCTTAAGGGGTTTGACAA TTTCGATGTAGATGCCTGCACGGCTCGCGG GGTATGGTTAACATTCGTCCCTGATCTCCT GACGGTTCCAACCGCGGAGCTGGCAATTG GCCTCGCGGTAGGTTTAGGGCGTCATCTG CGGGCAGCAGACGCATTCGTCCGCTCGGG CAAATTCCGTGGTTGGCAACCACGTTTCTA TGGTACAGGGCTTGATAATGCTACGGTAGG CTTCCTGGGTATGGGGGCCATCGGTCTTG CCATGGCAGACCGTCTTCAGGGGTGGGGC GCAACCTTACAGTATCATGAGGCTAAGGCT TTGGATACGCAGACAGAGCAGCGTCTCGG TCTGCGCCAAGTCGCGTGCAGCGAACTGTT CGCTAGTTCGGATTTTATTCTGCTTGCGTTA CCGCTCAATGCAGACACCTTGCATCTTGTA AACGCTGAACTTCTGGCCTTGGTACGGCCT GGCGCACTCCTCGTGAACCCTTGCCGCGG GAGTGTTGTTGATGAGGCCGCGGTGCTTG CTGCGCTTGAACGCGGCCAGCTGGGCGGC TACGCTGCCGATGTGTTTGAAATGGAAGAC TGGGCCCGCGCGGATCGTCCACAACAGAT TGACCCGGCGCTGCTTGCACATCCAAATAC GCTTTTCACTCCGCATATCGGCTCGGCGGT ACGCGCCGTACGTCTTGAGATTGAGCGGT GTGCTGCCCAGAACATTCTCCAAGCGCTGG CCGGCGAGCGTCCTATCAATGCAGTCAATC GCTTGCCGGGTGGTGGCAGCGGTGGGGG CTCGGGTGGTGGTTCTCATCACCACCATCA CCAT	MLPKLVITHRVHEEILQLLAPHCELITNQTDS TLTREEILRRCRDAQAMMAFMPDRVDADF LQACPELRVIGCALKGFDNFDVDACTARGV WLTFVPDLLTVPTAELAIGLAVGLGRHLRAA DAFVRSGKFRGWQPRFYGTGLDNATVGFL GMGAIGLAMADRLQGWGATLQYHEAKALD TQTEQRLGLRQVACSELFASSDFILLALPLN ADTLHLVNAELLALVRPGALLVNPCRGSVV DEAAVLAALERGQLGGYAADVFEMEDWAR ADRPQQIDPALLAHPNTLFTPHIGSAVRAV RLEIERCAAQNILQALAGERPINAVNRLPGG GSGGGSGGGSHHHHHH
PTDH_MxTP	ATGTTGCCTAAGCTTGTGATTACACATCGC GTACATGAGGAGATTCTGCAGTTATTGGCG CCTCATTGTGAGCTGATTACCAACCAGACA GATTCAACCCTCACGCGTGAAGAAATTTTA CGCCGTTGTCGTGACGCTCAAGCGATGAT GGCATTTATGCCGGACCGCGTTGATGCTGA CTTCTTACAAGCCTGTCCTGAACTGCGTGT AATCGGGTGCGCTCTTAAGGGGTTTGACAA TTTCGATGTAGATGCCTGCACGGCTCGCGG GGTATGGTTAACATTCGTCCCTGATCTCCT GACGGTTCCAACCGCGGAGCTGGCAATTG GCCTCGCGGTAGGTTTAGGGCGTCATCTG CGGGCAGCAGACGCATTCGTCCGCTCGGG CAAATTCCGTGGTTGGCAACCACGTTTCTA TGGTACAGGGCTTGATAATGCTACGGTAGG CTTCCTGGGTATGGGGGCCATCGGTCTTG CCATGGCAGACCGTCTTCAGGGGTGGGGC GCAACCTTACAGTATCATGAGGCTAAGGCT TTGGATACGCAGACAGAGCAGCGTCTCGG TCTGCGCCAAGTCGCGTGCAGCGAACTGTT CGCTAGTTCGGATTTTATTCTGCTTGCGTTA CCGCTCAATGCAGACACCTTGCATCTTGTA AACGCTGAACTTCTGGCCTTGGTACGGCCT GGCGCACTCCTCGTGAACCCTTGCCGCGG GAGTGTTGTTGATGAGGCCGCGGTGCTTG CTGCGCTTGAACGCGGCCAGCTGGGCGGC TACGCTGCCGATGTGTTTGAAATGGAAGAC TGGGCCCGCGCGGATCGTCCACAACAGAT TGACCCGGCGCTGCTTGCACATCCAAATAC	MLPKLVITHRVHEEILQLLAPHCELITNQTDS TLTREEILRRCRDAQAMMAFMPDRVDADF LQACPELRVIGCALKGFDNFDVDACTARGV WLTFVPDLLTVPTAELAIGLAVGLGRHLRAA DAFVRSGKFRGWQPRFYGTGLDNATVGFL GMGAIGLAMADRLQGWGATLQYHEAKALD TQTEQRLGLRQVACSELFASSDFILLALPLN ADTLHLVNAELLALVRPGALLVNPCRGSVV DEAAVLAALERGQLGGYAADVFEMEDWAR ADRPQQIDPALLAHPNTLFTPHIGSAVRAV RLEIERCAAQNILQALAGERPINAVNRLPGG GSGGGSGGGSPEKRLTVGSLRR

	GCTTTTCACTCCGCATATCGGCTCGGCGGT<br>ACGCGCCGTACGTCTTGAGATTGAGCGGT<br>GTGCTGCCCAGAACATTCTCCAAGCGCTGG<br>CCGGCGAGCGTCCTATCAATGCAGTCAATC<br>GCTTGCCGGGTGGTGGCAGCGGTGGGGG<br>CTCGGGTGGTGGTTCTCCAGAAAAGCGGC<br>TTACTGTGGGGTCGTTACGGCGT	

Supplementary Table 2. DNA primers used in this study.

Primer	Sequence (5'->3')
pETDuet_F	ATTAACCTAGGCTGCTGCCACCG
pETDuet_B1	TGTATATCTCCTTCTTATACTTAACTAATATACTAAGATGGGGAATTG
pETDuet_B2	GTATATCTCCTTCTTAAAGTTAAACAAAATTATTTCTAGAGGGG
Mx_pmut_F	CTTATATTCCCAGTTACATCGGGGTGGCGAGATCGAGACAATTCGCC
Mx_pmut_R	CCGATGTAACTGGGAATATAAGC
NanoLuc_His_F	AAGTATAAGAAGGAGATATACAATGGTTTTCACCTTAGAAGATTTCGTG
NanoLuc_His_R	GCAGCAGCCTAGGTTAATTCAATGGTGATGGTGGTGATGTGAACCGCCCCCGCTC
Adhd_His_F	AAGTATAAGAAGGAGATATACAATGAAGCGCGTTAAC
Adhd_His_R	GCAGCAGCCTAGGTTAATTCAATGGTGATGGTGGTGATGGCTACCACCCCCAGAAC
PTDH_His_F	TTAACTTTAAGAAGGAGATATACATGTTGCC
PTDH_His_R	GCAGCAGCCTAGGTTAATTCAATGGTGATGGTGGTGATGAGAACCACCACCCGAGC

Supplementary Table 3. Cryo-EM data collection, refinement, and validation statistics.

	Mx_pmut T=1 (EMDB-44383) (PDB 9B9I)	Mx_pmut T=3 (EMDB-44388) (PDB 9B9Q)	Mx_pmut T=4 (EMDB-44427) (PDB 9BC8)
**Data collection and processing**			
Magnification	45,000x	45,000x	45,000x
Voltage (kV)	200	200	200
Electron exposure (e–/Å^2)	39.18	39.56	39.56
Defocus range (μm)	-1.0 to -1.8	-1.0 to -1.8	-1.0 to -1.8
Pixel size (Å)	0.91	0.91	0.91
Symmetry imposed	I	I	I
Initial particle images (no.)	66,813	37,600	37,600
Final particle images (no.)	61,808	12,967	4,386
Map resolution (Å)	2.86	3.14	3.46
FSC threshold	0.143	0.143	0.143
**Refinement**			
Initial model used (PDB code)			
Model resolution (Å)	3.2	3.6	3.9
FSC threshold	0.5	0.5	0.5
Map sharpening *B* factor (Å^2)	-126.4	-93.1	-87.9
Model composition			
Non-hydrogen atoms	2,038	6,569	8,740
Protein residues	262	848	1,128
Ligands	0	0	0
B factors (Å^2)			
Protein	43.61	60.14	104.39
R.m.s. deviations			
Bond lengths (Å)	0.005	0.005	0.005
Bond angles (°)	1.093	1.039	1.095
Validation			
MolProbity score	2.09	2.30	2.04
Clashscore	8.64	11.77	13.43
Poor rotamers (%)	3.33	2.94	3.41
Ramachandran plot			
Favored (%)	96.51	94.86	93.97
Allowed (%)	3.49	5.14	5.94
Disallowed (%)	0	0	0.9
